# A New Medicine—“Seven Springs” “Iron and Alum Mass,” or “Compound Ferruginous Mass.”

**Published:** 1876-12

**Authors:** W. F. Barr

**Affiliations:** Abingdon, Va.; Member of the American Medical Association, one of the Vice Presidents of the Medical Society of Virginia, Ex-President and now Corresponding Secretary of the Abingdon Academy of Medicine—Honorary Member of the Medical Society of Wythe county, Va.—Physician of the Alms House and to the Prison of Washington county, Va., &c., &c.


### SOUTHERN MEDICAL RECORD.

VOL. VI. DECEMBER, 1876. NO. 12.

### Original Communications.

### A NEW MEDICINE—"SEVEN SPRINGS" "IRON AND ALUM MASS" OR "COMPOUND FERRUGINOUS MASS."

BY W. F. BARR, M. D., OF ABINGDON, VA.

[Member of the American Medical Association, one of the Vice Presidents of the Medical Society of Virginia, Ex-President and now Corresponding Secretary of the Abingdon Academy of Medicine—Honorary Member of the Medical Society of Wythe county, Va.—Physician of the Alms House and to the Prison of Washington county, Va., &c., &c.]

It has been remarked, and daily repeated, that "there is nothing new under the sun." This may be true, and it is equally true that it has taken many years before the mind of man, with all its powers of discovery and invention, has been enabled to obtain information on subjects that are very old. For instance Iron and Alum are *old* remedies, but *new* combinations have been frequently made, their therapeutical properties well ascertained, and their application to diseases been successfully tested. It is, therefore, unnecessary at the present time to write an essay either on the medicinal virtues and properties of Iron and Alum, or the mineral from which they are obtained; nor is it necessary or proper that time should be consumed in alluding to any changes which may have taken place in the blood. It is my purpose to contribute something *practical*, founded on correct, scientific principles long since established by experiments now unnecessary to repeat. Theory, reason, principle, are necessary "in their place," but "without experience all preconceived theory would be vain and useless." Works on Therapeutics teach the effects of Iron, Alum, Soda, Potash, Magnesia, and other Medicines contained in the one now under consideration.

I wish now to invite the attention of the profession to a new preparation or combination of these Medicines. It is known in this section of the State, in which it is manufactured, as "SEVEN SPRINGS IRON AND ALUM MASS," or "COMPOUND FERRUGINOUS MASS."

It is manufactured in Washington county, Virginia, from the waters of seven mineral springs, and is made by evaporation. The analysis of Professor J. W. Mallet, of the University of Virginia, which will be given below, finds it to contain Iron, Alum, Magnesia, Lime, Lithia, Nickel, Manganese, Copper and Zinc.

ANALYSIS BY PROF. J. W. MALLET, OF THE UNIVERSITY OF VA.

The Mass appears as a stiff dough, or soft solid, of light gray color, and marked acid reaction to test-paper. The contents of several bottles having been thoroughly mixed, the following composition was found for the mixture:

| IN 100 PARTS. | | IN 100 PARTS. | |
|---|---|---|---|
| Aluminum sulphate | 15.215 | Potassium sulphate | 060 |
| Ferric sulphate (per sul. iron) | 4.628 | Sodium sulphate | 226 |
| Ferrous sulphate (protosul. iron) | 412 | Lithium sulphate | 019 |
| Nickle sulphate | 162 | Ammonium sulphate | 022 |
| Cobalt sulphate | 014 | Sodium chloride | 326 |
| Manganese sulphate | 257 | Calcium fluoride | trace |
| Copper sulphate | 008 | Calcium phosphate | trace |
| Zinc sulphate | 301 | Silica | 1,504 |
| Magnesium sulphate | 16.006 | Organic matter | 123 |
| Strontium sulphate | trace | Water | 42.938 |
| Calcium sulphate | 17.538 | | |
| | | | 99.759 |

From the above analysis it will be observed that the "Mass" contains some of our best mineral tonics and the alkalies. It has been within the past few years extensively and successfully used in the treatment of different diseases by physicians throughout Virginia. It possesses tonic and alterative properties, and is applicable in all cases of nervous or muscular debility; and in which it is desired to improve an impoverished condition of the blood. It is, therefore, beneficial in dyspepsia, constipation, chronic bronchitis, rheumatism, neuralgia, chronic dysentary, erysipelas, anæmia, menorrhagia, dysmenorrhœa, an-

æmia and chlorosis and diseases following typhoid, intermittent and remittent fevers, chills and fevers or such diseases as are caused by malaria. But I stated I intended to contribute something practical. I will proceed to report some cases of interest treated and *cured* by this medicine.

CASE 1.—*Dyspepsia.*—Mrs. J., age twenty-six, delicate constitution, nervous and debilitated; acidity and burning of the stomach; pyrosis; tongue furred with yellow coating; but little appetite, pulse regular, but weak and frequent, bowels irregular, sometimes costive, at other times acted on several times a day; sleeps but little, suffers frequently with headache, catamenia irregular—returning at periods of four, five and six weeks, the discharge at one time being little, at another profuse. She had a miscarriage about one year ago. (May, 1875.) I had prescribed for her tincture chloride of iron, quinine, strychnine, bismuth, and good diet, such as would agree with her, regular out door exercise. Under this treatment she did not improve. I then prescribed for her the Iron and Alum Mass—a pill three times a day half hour after eating. After using this for five or six weeks, she was cured.

CASE II.—*Dyspepsia.*—Mrs. M., age fifty, nervous temperament, appetite generally good, tongue clean. with exception posterior portion slightly covered with yellow coating; rarely ever suffered with headache, acidity and burning sensation in the stomach, sour eructations, costive, pulse regular, seventy-six per minute, had been for several years suffering with cervical-endometritis, of which she had been relieved. I had given her an occasional dose of blue pill, followed by rhubarb or magnesia, carbonate of soda, phosphate of sodo, bicarb-potash, wine of pepsin, containing bismuth and nux vomica, Fowler's solution of arsenic, gentian and columba, but all to no effect. I then prescribed the Iron and Alum Mass, and also the waters of the springs, and she was *cured.*

CASE III.—"*Indigestion.*"—Mr. C., aged forty-seven. This gentleman complained of nothing but a feeling of fullness of the stomach after eating. His appetite was good, bowels regular, pulse regular, seventy-six per minute, slept well, kindneys and bladder acting well—in healthy condition, but after each meal he stated that he would feel as if a large lump was in his stomach. He commenced using the Iron and Alum Mass, which I directed him to continue, which he did, and he is now entirely relieved.

CASE IV.—*Sleeplessness.*—Rev. J. A., age twenty-six, nervous-bilious temperament, well educated, of sedentary habits, had not had any attack of sickness, but is of a delicate constitution, very nervous; was afraid of everything or any noise that could not be easily accounted for, which condition had existed and continued for about two years; used tobacco, smoked and chewed, appetite good but irregular, sometimes nervous, especially after getting sound sleep, tongue coated with yellow fur, (no sourness or acidity of the stomach) belching after eating and drinking water, fullness and heaviness of the stomach, difficulty of breathing, so much so, thought he had asthma; inclined to costiveness, which was prevented by exercise and *habit* of going to stool regularly; urinary organs in healthy condition. His chief trouble was *sleeplessness*, so much so that sleep only followed exhaustion or sleeping in strange rooms; pulse regular, seventy-six per minute, temperature natural. He had not taken any medicine with which to procure sleep, but had taken pepsin, strychnine and bismuth for dyspeptic symptoms. He was put on the use of Iron and Alum Mass, and after taking it for two or three weeks, was cured and remains so now. Six months since he was cured.

CASE V.—*Amenorrhœa.*—Miss——had her menses suppressed for several months; she had no particular symptoms of disease that required special treatment; appetite good, pulse regular, bowels regularly operated on. Prescribed Iron and Alum Mass, and after using it for a few week was relieved.

CASE VI.—*Irregular Menstruation.*—Miss——, nervo-bilious temperament, appetite good, pulse regular, bowels regular, well educated and refined, and, although she would at times, after slight exposure, become irregular in her menstruation, she would be "unwell" every two or three weeks, but after using the Iron and Alum Mass was in a short time relieved.

Case VII.—Mr. T. C., aged thirty-eight, entered the Confederate army at the age of twenty-three; was wounded 17th of March, 1863, the ball entering the epigastrium, making its exit left of the lower lumber vertrebra, near the kidneys; was unable to move for five days and nights; was very much debilitated for a year, although his general health was good, and returned to the army and reported for duty 22d May, 1864; was in an engagement or fight every day until the 28th day of May, when he received a gunshot wound in the left ankle joint, with a minnie ball, by an accidental shot from the gun of a member of the same company, in an engagement with the Federal troops at Hall's Shops, near Enon Church, Hanover county, Virginia. The ball entered the ankle and lodged in its anterior part. The foot was amputated at the ankle joint. He remained in Jackson hospital, near Richmond, until the following August, when he returned to his home near Abingdon. The stump did not heal, but would suppurate about once a month for about two years, afterwards, at intervals for several years; he remained debilitated from the suppuration and drainage of the stump, though he weighed one hundred and eighty pounds. For *eight years* all remedies failed to heal the stump. Different physicians, as well as myself, were called upon to treat the case. The pain in the stump was constant and at times violent, so much so that he requested me and others to amputate the leg above the ankle joint. I saw him frequently. At last he was put upon the use of the Seven Springs Iron and Alum Mass. He took a pill three times a day, and after drying and pulverizing some, would sprinkle it on the sore pretty freely twice a day, and over this applied an ointment similar to resin cerate (it being composed of tallow, beeswax and resin). In less than ten weeks the stump was entirely healed, and has remained so for four years, covered with sound, healthy skin. His general health is good; tongue clean, appetite excellent, bowels regular, skin of natural temperature, pulse regular, seventy per minute, and weighing about one hundred and eighty-six pounds, sleeps well, and entirely free from pain.

Case VIII.—I am indebted to Dr. G. W. Semples, of Hamp ton, Virginia, for reporting this case. From a letter dated May 27th, 1876, I take the liberty of making the following extracts: "I have used the remedy (Iron and Alum Mass) as a tonic and alterative in several cases to great advantage, finding it to correspond well in its therapeutical action to the description in the circular. The most beneficial use of it I have made was in the case of an infant who, after having had summer complaint, took syphilis from its nurse, which went on to tertiary symptoms, and left it the most emaciated and debilitated little creature I ever beheld. It checked the diarrhœa that seemed amenable to no other treatment, improved its appetite, and with the aid of lactopepsin, restored its digestion, and made a cure of a case which nothing but the unflagging faith of the mother induced me to treat." From a letter written by Dr. Semples, dated December 27, 1875, I take the liberty of making the following extract: "I have used the vial of Iron and Alum Mass with very great advantage. I gave it to ——— ——— and it acted most admirably. Her appetite, which had been *nil* for many months, was quickly improved and soon fully restored, and her constipated bowels became regular, whilst the urine, which had been generally loaded with uric acid, became clear, and whilst taking it she had no attack of nephritic colic, to which she had been subject. She was suffering from disease of the pesterior nares, with pharyngitis and slight laryngitis, which had grown no better from varied applications I had made, and many suggestions by Professor———. These, too, seemed to be much benefitted by its use as a gargle and to wash out the nares, but more, perhaps, by its effects on her appetite and nutrition. Suffering from cough and tendency to, or rather with symptoms of incipient tuberculosis scrofulosis, the cough has been greatly relieved, and the other symptoms seem to retrograde with the improvement in general health and increase of weight."

Dr. Semples is an experienced and talented physician, and all who know him will fully appreciate his opinion on any medical subject.

From a letter written by Dr. C. Hardy, of Columbus, Miss., I take the following extract:

"I regard the Seven Springs Iron and Alum Mass a very valuable medicinal agent. I know of no other remedy that combines more happily tonic, alterative and diuretic properties. I myself had been a victim to dyspepsia for many years, and the use of a few bottles has restored me to perfect health."

Dr. William White, an intelligent and well read physician of Abingdon, Virginia, writes:

"I have used the Iron and Alum Mass, as made from the waters of the Seven Springs, and cheerfully add my testimony of its value in scrofulous affections, and some of the diseases of females. I deem it unnecessary to give in detail the numbers of cases in which it has been used by me. In those cases of dysmenorrhœa when there is a functional disturbance of the organ, and dependent on the general condition of the health, as indigestion, constipation of the bowels, torpidity of the liver, &c., I have found it to be of much value."

Dr. E. M. Campbell, a talented and experienced physician of this place, of upwards twenty-five years practice, in a letter published in the *Virginia Medical Monthly*, of June, 1876, says:

"I regard it as a most valuable addition to our list of remedies. * * * I have seen many cases of a scrofulous nature, where the benefit derived from the use of the water and Mass made from the same, was most striking."

The Abingdon Academy of Medicine, of Abingdon, Washington county, Virginia, adopted the following resolution:

"The Fellows of this Academy of Medicine regard the waters of the Seven Springs as efficient and valuable, and the Mass manufactured therefrom as a valuable contribution to Materia Medica; and especially adapted to many diseases and conditions to which the accompanying analysis would indicate its application."

I deem it unnecessary to report any more cases, or add further testimony in favor of the great value of this new medicine. It may, however, be proper to state that it, like many other medicines, has a different effect on the system according to the size of the dose, taking small doses—from two to five grains—acts as an astringent, and in larger doses it will act as an aperient or purgative. In doses of from ten to thirty grains, it acts as a tonic, alterative and diuretic.

